# Sequence Specificity of BAL 31 Nuclease for ssDNA Revealed by Synthetic Oligomer Substrates Containing Homopolymeric Guanine Tracts

**DOI:** 10.1371/journal.pone.0003595

**Published:** 2008-10-31

**Authors:** April Marrone, Jack Ballantyne

**Affiliations:** 1 Graduate Program in Chemistry, Department of Chemistry, University of Central Florida, Orlando, Florida, United States of America; 2 National Center for Forensic Science, Orlando, Florida, United States of America; University of Helsinki, Finland

## Abstract

**Background:**

The extracellular nuclease from *Alteromonas espejiana*, BAL 31 catalyzes the degradation of single-stranded and linear duplex DNA to 5′-mononucleotides, cleaves negatively supercoiled DNA to the linear duplex form, and cleaves duplex DNA in response to the presence of apurinic sites.

**Principal Findings:**

In this work we demonstrate that BAL 31 activity is affected by the presence of guanine in single-stranded DNA oligomers. Specifically, nuclease activity is shown to be affected by guanine's presence in minimal homopolymeric tracts in the middle of short oligomer substrates and also by its presence at the 3′ end of ten and twenty base oligomers. G•C rich regions in dsDNA are known to cause a decrease in the enzyme's nuclease activity which has been attributed to the increased thermal stability of these regions, thus making it more difficult to unwind the strands required for enzyme access. Our results indicate that an additional phenomenon could be wholly or partly responsible for the loss of activity in these G•C rich regions. Thus the presence of a guanine tract *per se* impairs the enzyme's functionality, possibly due to the tract's bulky nature and preventing efficient progression through the active site.

**Conclusions:**

This study has revealed that the general purpose BAL 31 nuclease commonly used in molecular genetics exhibits a hithertofore non-characterized degree of substrate specificity with respect to single-stranded DNA (ssDNA) oligomers. Specifically, BAL 31 nuclease activity was found to be affected by the presence of guanine in ssDNA oligomers.

## Introduction

The extracellular nuclease from *Alteromonas espejiana*, BAL 31 comprises several nuclease activities designated as “slow” (S) and “fast” (F) depending upon the relative rates with which they catalyze the terminally directed hydrolysis of duplex DNA [1]. The displayed nuclease activity also includes hydrolysis of single-stranded DNA (ssDNA), cleavage of negatively supercoiled DNA to the linear duplex form, and cleavage of duplex DNA in response to the presence of apurinic sites [2], [3]. The use of BAL 31 is favored in molecular cloning techniques due to its stability upon extended storage and resistance to inactivation in the presence of high concentrations of salt or denaturing agents [1]. Applications that benefit from the use of BAL 31 include those that require the progressive removal of nucleotides from both termini of double-stranded DNA (dsDNA) [4], [5], complete digestion of ssDNA, restriction site mapping in DNA [3], [6] and the detection of lesions or distorted structures in duplex DNA [7].

Although BAL 31 activity with duplex DNA is relatively well characterized [2], [3], there appear to be no detailed reports of studies examining how the BAL 31 enzyme interacts with short single stranded linear polymers of DNA containing homopolymeric tracts. Though it has been noted that the exonuclease activity is hindered by the presence of G•C sequence motifs [2], [3] in dsDNA, there has been no such hindrance noted for ssDNA. Here we focus on how the BAL 31 enzyme degrades single stranded DNA containing homopolymeric tracts, using homogeneous and heterogeneous 10–20 base oligomers. Specifically we determined the efficiency of hydrolysis of short 10–20 base oligomers and whether the enzyme exhibits any sequence specificity. We found that homopolymeric guanine oligomers are not digested by BAL 31 and that the presence of short dG tracts in mixed sequence oligomers hinder BAL 31 enzyme activity.

## Results

### Hydrolysis of dN_10_

**Figure 1:**
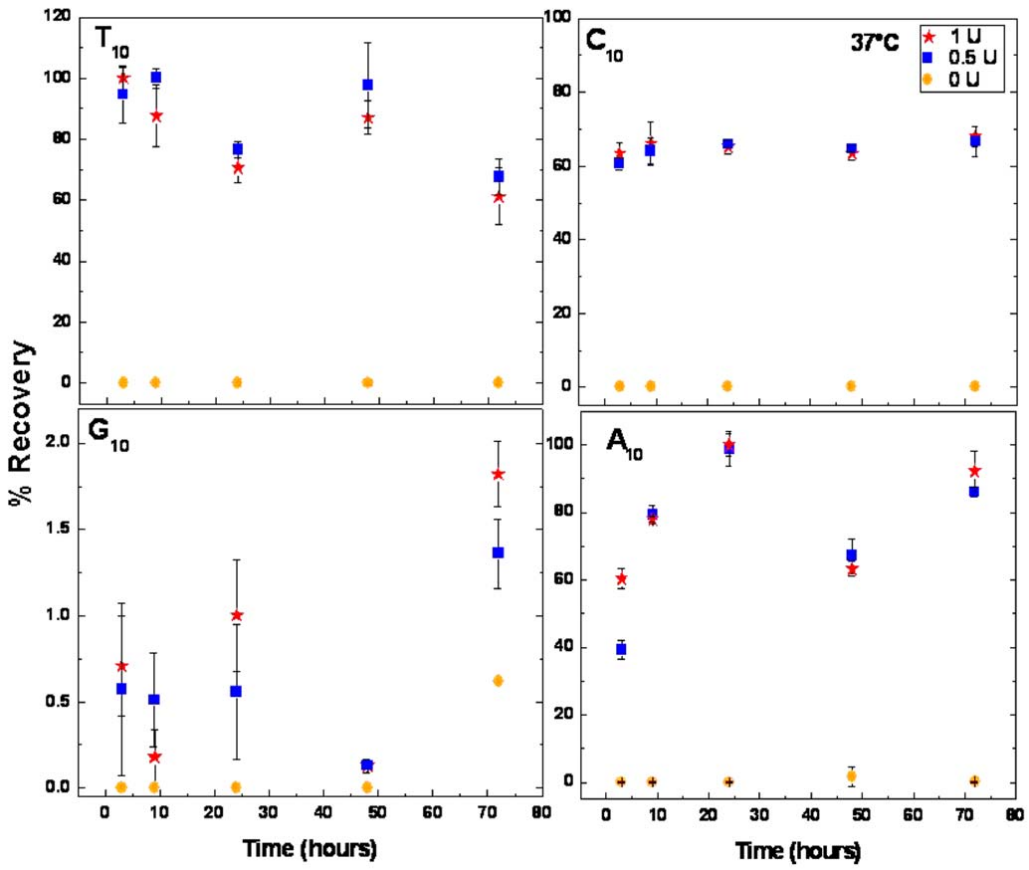
Hydrolysis of homodecameric oligomers. C_10_, A_10_, T_10_, and G_10_ were incubated at 37°C with 1 U, 0.5 U, and 0 U of BAL 31. The relative hydrolysis efficacy of each homopolymeric oligomer is indicated by the percentage recovery of their constituent 5′dNMPs over time (3–72 h). Incubation at 30°C gave similar results.

BAL 31 hydrolysis of the four dN_10_ homopolymers was carried out for varying periods (3, 9, 24, 48 and 72 h) at 37°C and with two different enzyme concentrations (0.5 and 1.0 U). Samples were prepared in triplicate. Oligomer hydrolysis was monitored quantitatively by the separation and detection of dNMPs by HPLC (Fig. 1). Of the four homopolymers the dT_10_ oligomer was, in general, the most efficiently hydrolysed substrate. DT_10_ incubated with 0.5 U BAL 31 resulted in ∼68–100% recovery of dTMP monomers, whereas with 1 U enzyme the dTMP recovery was ∼61–100%. The dC_10_ oligomer incubated with 0.5 U BAL 31 resulted in ∼61–67% recovery of dCMP monomers, whereas with 1 U enzyme the dCMP recovery was ∼63–68%. The dA_10_ oligomer incubated with 0.5 U BAL 31 resulted in ∼39–99% recovery of dAMP monomers, and with 1 U enzyme the dAMP recovery was ∼60–100%. In contrast to the other homopolymers, the dG_10_ oligomer proved to be highly refractory to hydrolysis by BAL 31. Specifically, the dG_10_ oligomer incubated with 0.5 U BAL 31 resulted in only 0.1–1.4% recovery of dGMP monomers, with 1 U enzyme the dGMP recoveries were ∼0.1–1.8%. The results were essentially the same for all four homoplymeric oligomers when the enzyme reaction took place at 30°C (data not shown).

**Figure 2:**
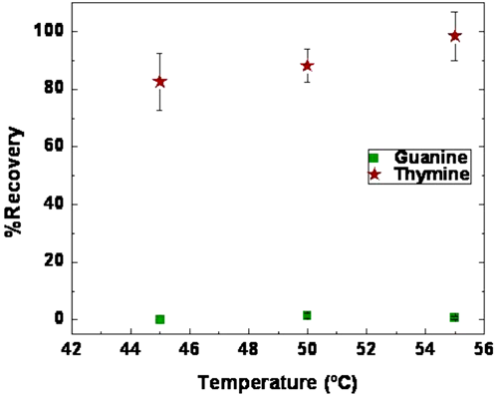
G_10_ homodecamers continue to be refractory to hydrolysis at elevated temperatures. G_10_ and T_10_ were hydrolyzed by BAL 31 at 45°C, 50°C, and 55°C for a twenty-four hour period. The relative hydrolysis efficacy of each homopolymeric oligomer is indicated by the percentage recovery of their constituent 5′dNMPs.

We considered the possibility that the dramatic reduction in hydrolysis efficiency with dG_10_ could be an artifact due to guanine homoploymer self aggregation to form higher order structures [8], [9] that may not be efficient substrates for the enzyme. Lowering the salt concentration or increasing the temperature might be expected to decrease such aggregation. However, lowering the salt concentration would be counter productive due to the requirement of Mg^2+^ and Ca^2+^ for enzymatic activity. Since the enzyme requires a large deactivation temperature (∼85°C) and presumably is still active at elevated temperatures, the enzyme reaction temperature was increased from 37°C to 45°C, 50°C, and 55°C and the dG_10_ oligomers were incubated over a twenty-four hour period with 0.5 U enzyme. DT_10_ oligomers were incubated under the same conditions as a control. Samples were prepared in quintuplet. The dT_10_ samples produced 82.6±9.9%, 88.2±5.9%, and 98.4±8.4% dTMP at 45°C, 50°C, and 55°C respectively. The dG_10_ samples produced 0±0%, 1.31±0.94%, and 0.78±0.46% dGMP at 45°C, 50°C, and 55°C respectively (Fig. 2). Even if not all secondary structure was eliminated at these elevated temperatures, it would be alleviated which should lead to an increase in dGMP produced. These results indicated that the refractory nature of dG_10_ to BAL 31 digestion was probably not due to the self aggregation of homoguanosine oligomers into higher order structures.

### Position and sequence requirements for the guanine inhibition of ssDNA by BAL 31

To further delineate the sequence length and position requirements for the dG_n_ mediated inhibition of BAL 31, a series of decamers were synthesized that (i) contained two guanines that ‘capped’ both the 5′ and 3′ ends (G_2_-CAP), (ii) contained a homopolymeric stretch of four guanines in the middle of the decamer (G_4_-MID), (iii) contained no guanine residues (G-NO), (iv) contained a homopolymeric stretch of four guanines at the 5′ end of the decamer (G_4_-5′CAP), and (v) contained a homopolymeric stretch of four guanines at the 3′ end of the decamer (G_4_-3′CAP). A series of 20-mers were also used that (i) contained a homopolymeric stretch of four guanines in the middle of the 20-mer (G_4_-MID-L), (ii) contained three guanines that ‘capped’ both the 5′ and 3′ ends (G_3_-CAP-L), and (iii) contained four guanines that ‘capped’ both the 5′ and 3′ ends (G_4_-CAP-L).

**Figure 3:**
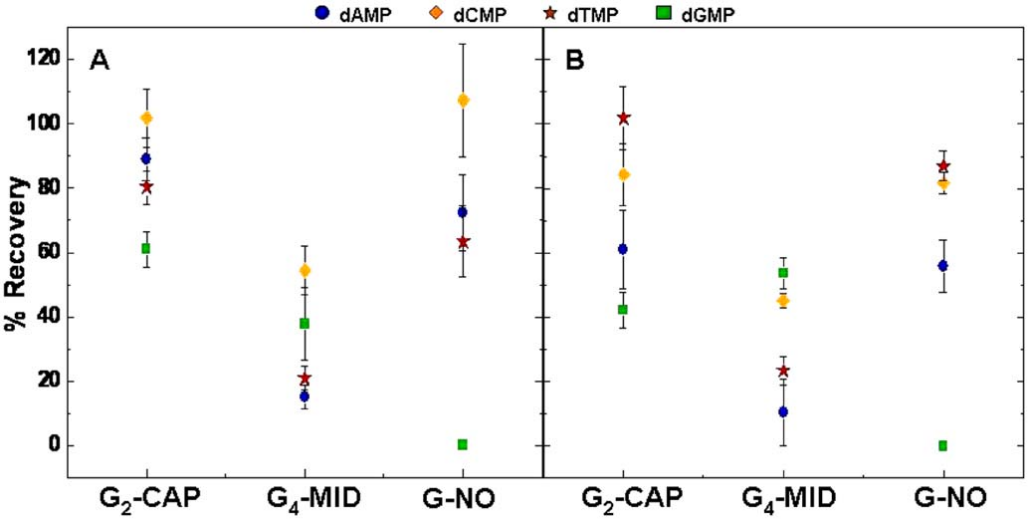
Hydrolysis efficiency of oligonucleotide substrates is affected by the location of the guanine tract. The relative hydrolysis efficacy of each oligomer is indicated by the percentage recovery of their constituent 5′dNMPs. (A) 300 µM oligomer substrates containing either no guanine (G-NO) or guanine tracts at the ends (G_2_-CAP ) or in the middle (G_4_-MID) of the polynucleotide chain were incubated at 37°C for twenty-four hours with 0.5 U BAL 31 enzyme. (B) The same experiment as in (A) except using 30 µM oligomers and an incubation temperature of 55°C.

The oligomers G-NO, G_2_-CAP, and G_4_-MID were incubated at 37°C with 0.5 U enzyme for a twenty-four hour period (in quintuplet) and, as before, their hydrolysis efficiency was measured by the recovery of the constituent nucleotides. An ANOVA analysis was conducted to determine whether the variation in recovery rates were significant. The G_2_-CAP oligomer produced nucleotides at recovery rates similar to those seen with the non-G containing homopolymeric oligomers and with the G-NO oligomer (Fig. 3A). In contrast, G_4_-MID produced non-G nucleotides at significantly lower recovery rates compared to G_2_-CAP. G-tract hydrolysis, as measured by the formation of dGMP, occurred with both G_2_-CAP and G_4_-MID, but at lower amounts than the other three nucleotides. Thus G di-nucleotides at the 5′ and 3′ ends of decameric oligonucleotides appear to be permissive for BAL 31 digestion of the decamer, whereas a G tetra-nucleotide tract in the middle of the decamer makes it more refractory to hydrolysis. This possibly indicates that the hydrolysis proceeds from one end of the ssDNA and/or that guanine tracts are difficult to fully hydrolyze.

The above experiments used oligomers at a concentration of 300 µM and the hydrolysis proceeded at 37°C. In order to preclude the possibility that self aggregation of the G_4_-MID oligomer [8], [9] was responsible for the observed hindrance of enzymatic activity, the reactions were repeated in quintuplet using conditions that would reduce the possibility of self-aggregation. Specifically, hydrolysis proceeded using a ten fold less concentration of oligomer substrates (30 µM) and at an elevated temperature (55°C). The results obtained were similar to those found at the lower temperature and higher concentration, thus providing support for the hypothesis that self-aggregation is not the cause of the decreased digestion rate with tracts of G residues (Fig. 3B). In order to preclude the possibility that the aberrant enzyme activity noted with different substrates was due to peculiarities attached to one particular batch of enzyme (such as co-contaminants etc), some of the experiments were repeated using BAL 31 nuclease from a different manufacturer (USB Corporation, Cleveland, OH, USA). These additional experiments included the digestion of dT_10_, dG_10_, G-CAP, G-MID, and G-NO at temperatures of 37°C and 55°C using 0.5 U enzyme over a twenty-four hour incubation period. The results were essentially the same as that obtained with the New England Biolabs enzyme used in the initial studies (data not shown) indicating that the refractory nature of guanine tracts to BAL 31 digestion was an inherent property of the enzyme. Moreover, the USB Corporation purified the enzyme using SDS-PAGE and reported only seeing two bands corresponding to the ‘fast’ and ‘slow’ forms of the enzyme. In order for the activity seen to be due to a contaminant, the contaminating species would have had to have been of similar size as one of the species comprising the BAL 31 enzyme and co-elute as such.

Further studies were carried out using the oligomers G_4_-5′CAP, G_4_-3′CAP, G_4_-MID-L, G_3_-CAP-L and G_4_-CAP-L to see if further insight could be obtained into the mechanism of action of BAL 31. For all five oligomers the mean recovery of each nucleotide after BAL 31 hydrolysis is shown in Table 1. An ANOVA analysis was conducted to ascertain the significance of the differences observed. According to the least significant difference, recovery of dCMP from G_3_-CAP-L was the same as from the other four oligomers (G_4_-5′CAP, G_4_-3′CAP, G_4_-MID-L, G_4_- CAP-L). G_4_-5′CAP was statistically the same as G_4_-3′CAP and G_3_-CAP-L, where G_4_-3′-CAP was additionally similar to G_4_-MID-L. G_4_-CAP-L, G_3_-CAP-L, and G_4_-MID-L had the same amount of dCMP recovery according to statistical analysis. Recovery of dAMP from G_4_-CAP-L, G_4_-5′CAP, G_3_-CAP-L, and G_4_-MID-L did not differ significantly and recovery from G_4_-3′CAP and G_4_-MID-L did not differ significantly. In addition G_4_-MID-L did not differ from G_4_-3′CAP. G_3_-CAP-L and G_4_-MID-L do differ significantly. The recoveries of dTMP from G_4_-CAP-L, G_4_-MID-L, and G_4_-5′CAP were statistically indistinguishable, and recovery from G_4_-5′CAP was similar to G_3_-CAP-L. Recovery from G_4_-3′CAP differed from the other four oligonucleotides. Most importantly, recovery of dGMP differed significantly between all oligomers except G_4_-CAP-L and G_3_-CAP-L.

**Table 1:** Recovery of dNMPs after BAL 31 hydrolysis.

Oligonucleotide	% dAMP	% dCMP	% dTMP	% dGMP
G_4_-5′CAP (5′-GGGGATCTCA)	66.1±6.4	100±4.9	100±8.0	29.3±4.5
G_4_-3′CAP (5′-ATCTCAGGGG)	46.2±1.0	97.5±2.2	56.5±1.5	100±2.7
G_4_-MID-L (5′-ATCTCATCGGGGTCATCTCA)	52.8±4.6	85.4±5.5	86.4±5.5	74.1±3.4
G_3_-CAP-L (5′-GGGATCTCATCTCATCTGGG)	74.3±6.7	96.0±9.7	115±10.0	53.0±4.8
G_4_-CAP-L (5′-GGGGTCTCATCTCATCGGGG)	64.4±4.6	75.5±2.4	85.6±3.7	41.3±1.3

The decamer that has four guanines at the 5′ end (G_4_-5′CAP) was refractory to G tract hydrolysis whereas, in contrast, almost complete recovery of dGMP occurred with the decamer with four guanines at the 3′ end (G_4_-3′CAP). Surprisingly, the hydrolysis of the other non-G nucleotides was more efficient with G_4_-5′CAP than with G_4_-3′CAP. This data supports the notion that, independent of any endonuclease activity it may possess, the BAL 31 acts as a ssDNA 3′→5′ exonuclease particularly when the substrate is of minimal length (10 mer) as previously reported with single stranded viral ΦX174 (wild type) DNA[10]. With the longer 20-mer substrates, recovery of dGMP was greater when the G tract was in the middle (G_4_-MID-L) as opposed to being capped at both ends (G_3_-CAP-L and G_4_-CAP-L). While ostensibly mimicking its shorter counterpart G_4_-MID, the G_4_-MID-L 20-mer, in contradistinction to its shorter counterpart G_4_-MID, was not more refractory to hydrolysis than its capped end homologs (G_3_-CAP-L , G_4_-CAP-L). Thus an increase in the length of the polynucleotide chain from 10 to 20 appeared to overcome the G-tract mediated inhibition of hydrolysis when the G tract was in the middle of the polynucleotide chain.

## Discussion

This study has revealed that the general purpose BAL 31 nuclease commonly used in molecular genetics exhibits a hithertofore non-characterized degree of substrate specificity with respect to ssDNA oligomers. Specifically, BAL 31 nuclease activity was found to be affected by the presence of guanine in ssDNA oligomers and the subsequent use of different G-tract containing substrates allows us to speculate on the likely mode of action of the enzyme. Minimal G tracts of four bases in the middle of short oligomers and at the 5′ end of decamers are refractory to hydrolysis. The enzyme does not appear to ‘skip over’ the difficult to digest tracts of guanine but appears to be hindered by them resulting in a loss of processivity momentum. Previous reports demonstrating that high G•C content in dsDNA hindered digestion [3], [11] hypothesized that this may be due to the greater difficulty in unwinding the thermodynamically more stable G•C rich DNA, a necessity for phosphodiester bond cleavage. However our data suggest an alternative explanation for the refractory nature of guanine rich regions of ssDNA to BAL 31 digestion and this may also pertain in part to the resistance of G•C rich regions of dsDNA.

The lack of digestion of the dG_10_ oligomer indicated an inhibition of the nuclease's activity when guanine is encountered. When guanine was present as a two base tract at the 5′ and 3′ ends of a decamer the result was the almost complete digestion of the adenine, thymine, and cytosine interstitial nucleotides, yet only a little over half of the guanine nucleotides were recovered. This result is consistent with a mechanism in which nucleotide hydrolysis begins at one end of the polynucleotide strand, as previously reported [10], [12], including the first two guanines, then proceeds in a processive manner along the strand digesting the next six nucleotides, but then possessing insufficient momentum to “push through” the last two guanines. When guanine was present in a four base sequence in the middle of a decamer, approximately half or less of all bases were recovered as their respective 5′-mononucleotides. This too can be explained by digestion being hindered when encountering the guanine homopolymeric four base stretch. When four guanines were positioned at the 5′ end of a decameric oligomer the non-G nucleotides were hydrolysed rather efficiently, yet less then 30% of the guanine mononucleotides were recovered. This is consistent with digestion beginning at the 3′ end and again being hindered when the four guanine tract was encountered at the other end of the polynucleotide chain. In contrast, when the same decameric oligomer was fashioned where the four guanine tract was positioned at the 3′ end, all of the guanines were digested, yet the recovery of dAMP and dTMP was decreased. This is also consistent with the enzyme encountering a loss of processive momentum due to the initially resistant guanine tract. When the four guanine tract was located in the middle of a twenty base oligomer approximately 74% of the guanine nucleotides were hydrolyzed. When there were three or four base guanine tracts at each end of a twenty base oligomer approximately 53% and 41% of the guanines were recovered, respectively. Again this result is consistent with hydrolysis of the initial guanine tract but being ‘slowed down’ in the process with the resulting processive momentum loss such as to lessen the ability of the enzyme to digest the last tract of guanines encountered at the other end of the oligomer. The recovery of the other three 5′-mononucleotides from G_4_-MID-L was lower than for G_3_-CAP-L and G_4_-CAP-L in general, which is consistent with the enzyme efficiently loading at one end and catalyzing hydrolysis of the non-G nucleotides at that end but subsequently being hindered in its processive track once it encounters the four base G tract in the middle of the oligomer. It is worth noting that Lu and Gray [12] provided evidence that removal of mononucleotides from very short oligomers (∼3 bases) may not be solely processive.

It is unclear why the presence of guanine tracts in ssDNA oligomers hinders BAL 31 hydrolysis activity. It is noted, however, that guanine is the bulkiest of the four nucleotides, since it is purine based and possesses two exocyclic functional groups (a primary amine and carbonyl) compared with the single functional group on the other purine nucleotide, adenine. Thus one hypothesis is that the active site of the enzyme is spatially constrained in such a manner that the bulky guanine tracts are bound and catalyzed more inefficiently than the other nucleotides. The resulting loss of ‘processive momentum’ could explain the activities noted in this study.

Future studies could include the use of nucleotide analog substrates containing a variety of different exocyclic architectures to permit further testing of the postulated hypothesis of enzyme inhibition by bulky nucleotides. Such studies would also provide a more precise delineation of the steric impediments to efficient catalysis. Further characterization of the noted ssDNA nuclease activity would include determining, after chromatographic fractionation, whether the activity was present in the F and/or S isoforms.

## Materials and Methods

### Sample preparation

Single stranded oligomers (dT_10_, dC_10_, dA_10_, dG_10_, 5′-GGATCATCGG (G_2_-CAP), 5′-ATCGGGGATC (G_4_-MID), 5′-ATATCATCAC (G-NO), 5′-GGGATCTCATCTCATCTGGG (G_3_-CAP-L), 5′-GGGGTCTCATCTCATCGGGG (G_4_-CAP-L), 5′-GGGGATCTCA (G_4_-5′CAP), 5′-ATCTCAGGGG (G_4_-3′CAP) and 5′-ATCTCATCGGGGTCATCTCA (G_4_-MID-L)) were cartridge purified (Invitrogen, Carlsbad, CA, USA). The deoxycytidine 5′-monophosphate (dCMP), thymidine 5′-monophosphate (dTMP), deoxyadenosine 5′-monophosphate (dAMP), and deoxyguanosine 5′-monophosphate (dGMP) standards were obtained from Sigma Aldrich (St. Lois, MO, USA). The nuclease BAL 31 was purified from the culture medium of *Alteromonas espejiana* BAL 31 containing a mixture of “fast” and “slow” species (New England BioLabs, Ipswich, MA, USA). Quality control assays and double stranded endonuclease activity were checked for by the manufacturer.

Oligomer samples were prepared by aliquoting the necessary volume to produce 6.0 nmol quantities (unless otherwise noted) into 1.5 ml microcentrifuge tubes and dehydrating in a vacuum centrifuge. Reactions were carried out in a 20 µl reaction volume containing 300 µM ssDNA oligomer, 20 mM Tris-HCl (pH 8.1 at 25°C), 600 mM NaCl, 12 mM CaCl_2_, and 12 mM MgCl_2_. Samples were incubated at 37°C for 24 hours unless otherwise indicated. Enzyme deactivation was accomplished by incubation at 95°C for 15 minutes.

### Sample analysis

Samples were analyzed using ion-pairing HPLC. The HPLC apparatus consisted of a SpectraSystem P2000 pump and a UV6000LP diode array detector (ThermoElectron, Waltham, MA, USA), equipped with a 5 cm light-path flow cell and data was collected between 200 and 300 nm. Data were acquired and analyzed by a PC using the XCalibur® software package provided by the HPLC manufacturer. Separation of the nucleotides was carried out using a Pinnacle II 250×4.6 mm, 5 µm particle size C_18_ column with a 10×2.1 mm guard column (Restek Corporation, Bellefonte, PA, USA). The ion-pairing technique was employed using buffers described by Tavazzi et al [13]. Buffer A (10 mM KH_2_PO_4_, 0.125% methanol, 12 mM tetrabutyl ammonium hydroxide, pH 7.0), and buffer B (100 mM KH_2_PO_4_, 30% methanol, 2.8 mM tetrabutyl ammonium hydroxide, pH 5.5) were used in a 50∶50 (v∶v) isocratic combination unless otherwise stated. A flow rate of 1.0 ml/min was maintained constant throughout the analysis and the analysis was conducted at ambient temperature (∼22°C). The use of HPLC to detect mononucleotides released by BAL 31 has been used previously with an alkaline salt gradient [10], however; the method took longer to elute all four dNMPs and the elution peaks were not well defined.

### Molecular species identification and statistical analysis

Molecular species identification was determined by matching retention times and absorption spectra to prepared standards. The peak areas of hydrolysis products obtained from HPLC-UV absorption measurements were quantified using the program XCalibur® applying an Avalon algorithm of peak detection.

The recovery percentage was calculated by dividing the number of moles of nucleotide recovered by the maximum number of nucleotide moles possible and multiplying by 100%. For example, 6 nmol of a 10-oligomer will theoretically yield a possible 60 nmol of nucleotides. Statistical analysis of data was carried out using ANOVA analysis where the between sample and within sample variances were compared using a one-sided F-test [14].
